# A Monolithic CMOS Magnetic Hall Sensor with High Sensitivity and Linearity Characteristics

**DOI:** 10.3390/s151027359

**Published:** 2015-10-27

**Authors:** Haiyun Huang, Dejun Wang, Yue Xu

**Affiliations:** 1 School of Electronic Science and Technology, Faculty of Electronic Information and Electronic Engineering, Dalian University of Technology, Dalian 116024, China; 2 Institute of New Electron Devices, Hangzhou Dianzi University, Hangzhou 310018, China; 3 College of Electronic Science & Engineering, Nanjing University of Posts and Telecommunications, Nanjing 210003, China; E-Mail: yuex@njupt.edu.cn

**Keywords:** monolithic Hall sensor, sensitivity and linearity, Hall offset, dynamic offset cancellation technique

## Abstract

This paper presents a fully integrated linear Hall sensor by means of 0.8 μm high voltage complementary metal-oxide semiconductor (CMOS) technology. This monolithic Hall sensor chip features a highly sensitive horizontal switched Hall plate and an efficient signal conditioner using dynamic offset cancellation technique. An improved cross-like Hall plate achieves high magnetic sensitivity and low offset. A new spinning current modulator stabilizes the quiescent output voltage and improves the reliability of the signal conditioner. The tested results show that at the 5 V supply voltage, the maximum Hall output voltage of the monolithic Hall sensor microsystem, is up to ±2.1 V and the linearity of Hall output voltage is higher than 99% in the magnetic flux density range from ±5 mT to ±175 mT. The output equivalent residual offset is 0.48 mT and the static power consumption is 20 mW.

## 1. Introduction

Hall device as a key component of contactless sensors for the detection of linear position, rotation angle, speed, and current, *etc.*, have been widely used in the fields of industrial control, consumer electronics, and the automotive industry [1,2,3]. They are usually integrated with a bias circuit, a read and interface circuit, an offset and noise elimination circuit, and a temperature stability circuit in a single chip, by increasing the system complexity to expand the functions of Hall sensor and improve the reliability [4,5,6]. Nowadays, monolithic integrated complementary metal-oxide semiconductor (CMOS) Hall sensors are greatly in demand due to the significant advantages such as high reliability, low power, and low cost. Unfortunately, the high doping level and shallow depth of the N-well active area lead to low magnetic sensitivity for the CMOS integrated Hall devices [7,8]. At the same time suffering from mask misalignment, non-uniformity distribution of impurities in active area, and influence of packaging stress *etc*., the offset voltage of CMOS integrated Hall devices is therefore very high [8,9,10]. For two kinds of Hall devices, namely horizontal Hall devices (HHDs) and vertical Hall devices (VHDs), the VHDs suffer lower magnetic sensitivity and higher offset than HHDs due to serious short circuit effects. Accordingly, the VHDs are generally fabricated in high-voltage (HV) CMOS technology with a deep N-well to reduce the short circuit effect, named HV-VHD [11]. However, the production cost of Hall sensors in HV CMOS technology remains relatively high. For this reason, Pascal *et al.* devised a shallow N-well VHD with high resolution using low-voltage standard CMOS technology, called LV-VHD [12]. In this device, the external contacts, *i.e.*, sensing contacts are situated outside the active region so that the short circuit effect is strongly suppressed. As a result, the offset and 1/f noise are considerably reduced, although the magnetic sensitivity remains low.

The well-known dynamic spinning current technique is very efficient for HHDs and HV-VHDs to remove the offset and 1/f noise [10,13]. It is worth to note that in the LV-VHDs, the Hall voltage is readout from the external contacts, which makes the role of the traditional two-phase spinning current circuit not obvious for offset and 1/f noise elimination [14]. On the other hand, the traditional spinning current circuit cannot provide a stable quiescent output voltage to ensure reliable operation of the follow-up amplifier [10,15]. In addition, there usually needs a more complicated signal conditioner following the spinning current circuit [15,16].

Presently, many techniques have been proposed to improve the performances of an integrated Hall sensor microsystem. Osberger *et al.* proposed a practical way to lower the 1/f noise and improve the resolution for LV-VHDs using a four-phase bi-current spinning current technique [14,17]. They showed that this spinning current technique not only sufficiently lowers the 1/f noise, but also efficiently reduces the non-linear offset caused by the PN junction field effect. A resolution of 37 μT over 1.6 kHz bandwidth and a residual offset of 0.1 mT were obtained for a LV-VHD senor fabricated in 0.35 μm standard CMOS technology, which is comparable to the performances of HHDs [17]. Heidari *et al.* recently presented a current-mode Hall magnetic sensor microsystem. Compared with the conventional voltage-mode Hall sensor, the current-mode Hall sensor provides a differential output Hall current signal to perform the current-mode signal processing and meanwhile uses the current spinning technique to cancel out the offset [18]. Consequently, a low residual offset of 50 μT and an extremely low power consumption of 120 μW were achieved by means of 0.18 μm standard CMOS technology [18].

In this paper, the horizontal Hall sensor microsystem with high sensitivity and high linearity is studied by optimizing the Hall plate structure and improving the traditional spinning current technique. We present a 5-V monolithic linear CMOS Hall sensor using dynamic spinning current offset cancellation technique. A cross-like Hall plate (horizontal Hall device) with long-contact structure is used to reduce the offset voltage from mask misalignment in the manufacturing process. By optimizing the ratio of finger length to finger width (*L/W*), the voltage related sensitivity (*S_V_*), and current related sensitivity (*S_I_*) of the cross-like Hall plate are improved simultaneously. In addition, a novel two-phase spinning current circuit is proposed to stabilize the quiescent output voltage in 1/2V_DD_. Based on 0.8 μm HV CMOS process, a monolithic linear Hall sensor integrated with an on-chip switched Hall plate and an efficient signal conditioner for offset cancellation and signal amplification has been fabricated and tested.

## 2. High Sensitive Hall Plate

### 2.1. Offset Reduction

**Figure 1:**
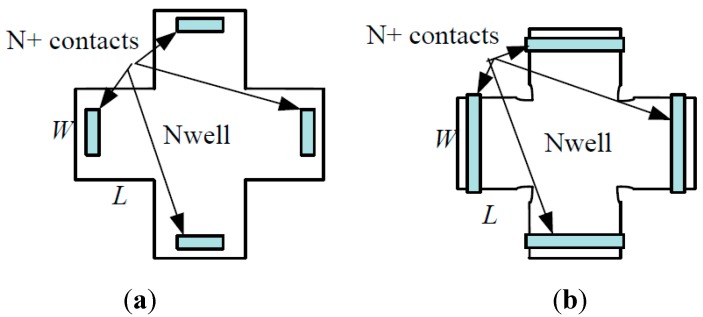
Cross-like Hall plate. (**a**) Conventional short contact structure; (**b**) Long contact structure.

Among these offset origins mentioned above, the offset coming from the mask misalignment can be reduced by optimizing the layout of Hall device. According to the principle of conformal mapping, a Hall device with smaller contact can obtain higher geometrical factor *G*, which is beneficial to improve the magnetic sensitivity. Therefore, the traditional Hall device generally applies the short contact structure, and the contacts are confined inside the Nwell active area, as shown in Figure 1a. But in the actual manufacturing process, there is misalignment between the N+ contacts and the Nwell masks. The shorter contact structure more easily gives rise to a larger misalignment, generating a greater Hall offset [8]. In order to reduce the offset caused by contacts shifting or rotating relative to Nwell, the layout design rule is deliberately violated in our work. The four fingers of the cross-like Hall plate are completely covered by four long N+ contacts, as shown in Figure 1b. Consequently, when the long contacts shift relative to Nwell, they can always cover the fingers of the Hall plate, which avoids the larger offset generation. But the long contact structure will cause the degradation of magnetic sensitivity, thus we need to further improve magnetic sensitivity of the cross-like Hall plate by optimizing device size and structure.

### 2.2. Sensitivity Improvement

The device geometrical factor *G* directly determines the magnetic sensitivity of the cross-like Hall plate. The correlation between the current related sensitivity (*S_I_*) and the geometrical factor *G* is a linear relationship, which is given by [8]:

(1) $$S_{I}\propto G=1-1.045e^{-\frac{\pi L}{W}}\cdot \frac{\theta_{H}}{\tan(\theta_{H})}$$

with $\theta_{H}$ the Hall angle defined by $\theta_{H}=\tan^{-1}(\mu_{H}B)$. $\mu_{H}$ is the Hall mobility and *B* is the magnetic flux density.

It is found that the *S_I_* can be improved by increasing the ratio of finger length to finger width (*L/W*). However, with the increase of *L/W* ratio, the input resistance of the cross-like Hall plate is also increased. The voltage related sensitivity (*S_V_*) can be calculated by the *S_I_* divided by the input resistance *R_in_* [8]:

(2) $$S_{V}=\frac{S_{I}}{R_{in}}\propto \frac{G}{2\frac{L}{W}+\frac{2}{3}}$$

where the input square resistance is the sum of the central region resistance (2/3) and two fingers resistances (2*L/W*).

It is obvious that the *S_V_* will be reduced with the *L/W* ratio increasing. For the long finger structure (*L* > *W*), it is equivalent to an increase of the resistivity of the finger connected to the bias voltage, which leads to the *S_V_* reduction. Therefore, we must consider the influence of the *L/W* ratio on the *S_V_*. To improve the *S_V_* and meanwhile not to decrease the *S_I_* too much, we optimized the device *L/W* ratio and structure. By appropriately reducing the finger length, the input resistance is reduced markedly, resulting in the increase of *S_V_*. Fortunately, the decrease of *S_I_* is small. On the other hand, due to lateral diffusion, the doping level of the narrow finger is lower than that of the central region, which increases the input resistance. Therefore, on the premise of maintaining the optimal *L/W* ratio, we increased the width of the fingers to obtain the uniform doping level across the whole Nwell implantation region. Furthermore, we etched four small notches on the four intersections between the fingers and central regions, as shown in Figure 1b. After diffusion, the Nwell implantation area is increased, which can result in a more homogenous doping level cross the central and finger regions.

### 2.3. Three-Dimension (3D) Device Simulation

According to 0.8 μm high voltage (HV) CMOS process parameters, we first performed two-dimension (2D) process simulation using Silvaco Athena software. With the aid of the 2D process simulation, we can obtain the impurity Gaussian distribution in the Nwell. The depth of the Nwell is about 5.5 μm. There appears a maximum impurity concentration of about 1.5 × 10^16^ cm^−3^ near the surface of N-well. In addition, we can obtain the doping level and the thickness of N+ contacts, which are about 1 × 10^20^ cm^−3^ and 0.3 μm, respectively. In terms of this key device process information provided by the process simulation, 3D device simulation of the cross-like Hall plate was carried out to obtain the offset voltage and magnetic sensitivity using Atlas device simulation tool. In the 3D device simulation, the physical models including carrier transport in an applied magnetic field, Shockley-Read-Hall (SRH) and Auger recombination, *etc*., were taken into account.

**Figure 2:**
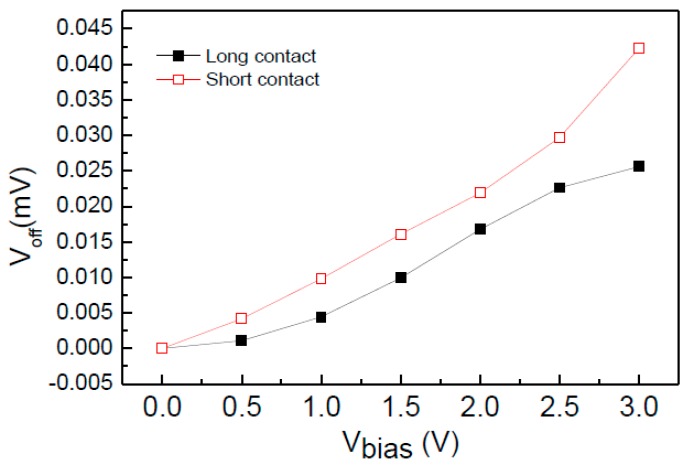
Offset voltage comparison of long-contact and short-contact Hall plates.

First of all, all the contacts of the two same cross-like Hall plates (*L* = 10 μm, *W* = 20 μm) with different contact lengths shift left 0.5 μm distance. The contact lengths of two Hall plates are 20 μm and 10 μm, respectively. The contact width of two Hall plates is 2 μm. The simulated offset voltage in the absence of the magnetic field is shown in Figure 2. It is clearly seen that there is a much smaller offset voltage in the long contact Hall plate. At 3 V biasing voltage, the offset voltage of the short contact Hall plate is 42 mV, while the offset voltage of the long contact Hall plate is only 25 mV. Therefore, the contacts completely covering the finger region can effectively reduce the offset voltage caused by the misalignment between the contacts and the Nwell.

**Figure 3:**
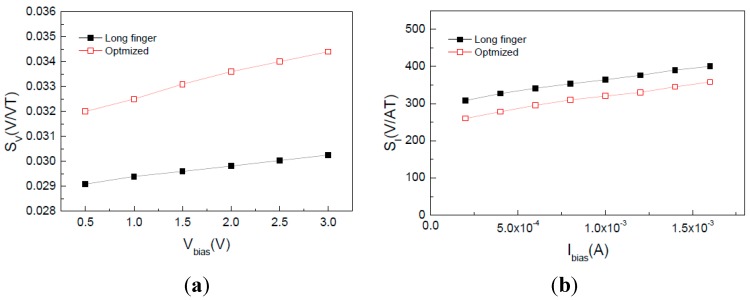
Magnetic sensitivity comparison of long-finger and optimized Hall plates. (**a**) *S_V_*; (**b**) *S_I_*.

Based on the long contact structure, we optimized the device structure parameters with the aid of 3D device simulation. When the *L/W* ratio of the cross-like Hall plate is decreased from long finger structure 2:1 (20 μm/10 μm) to 1:1 (20 μm/20 μm), and the notch in the cross intersections is 2 μm, we can achieve the optimal *S_V_* and *S_I_*. Figure 3 compares the *S_V_* and *S_I_* between the optimized and long finger Hall plates. It can be found that for the optimized Hall plate, the *S_V_* is significantly increased from 0.03 V/VT to 0.035 V/VT at 3 V bias voltage. In contrast, the *S_I_* is reduced from 360 V/AT to 320 V/AT at 1 mA biasing current. The reduction of *S_I_* is relatively smaller.

## 3. System Design and Simulation

**Figure 4:**
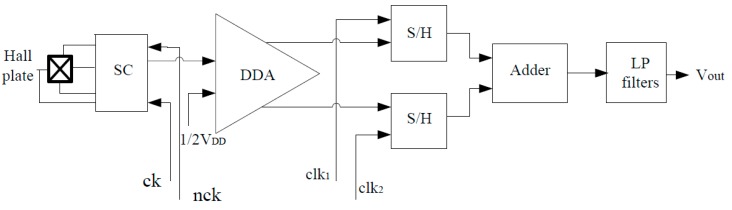
Block diagram of CMOS integrated linear Hall sensor microsystem.

Figure 4 shows a simplified block diagram of the CMOS integrated linear Hall sensor microsystem. The dynamic offset cancellation is implemented by two-phase spinning current technique [8,10]. Firstly, a low frequency Hall signal is modulated into the high frequency domain by a two-phase spinning current (SC) circuit that is controlled by a pair of complementary clocks ck and nck, respectively. At each clock state change, the Hall signal changes its polarity, while the polarity of the offset voltage is unchanged. After the spinning current modulation, the mixed signals including the Hall signal and offset are amplified by instrumentation amplifier (IA) simultaneously with two-sides output. Then, these two output signals of the instrumentation amplifier, corresponding to the first and second phase of spinning current course respectively, are sampled and held by two sample/hold (S/H) circuits. In order to avoid sampling at each plate switching, two narrow pulses clk_1_ and clk_2_ determine the sampling time of S/H circuits. It is worth noting that the polarity of Hall voltage hold by two S/H circuits is the same, while the polarity of offset voltage hold by two S/H circuits is the opposite. Following this, the outputs of the two S/H circuits input to the adder circuit to complete the demodulation function, thereby eliminating the offset signal and recovering the low frequency Hall signal. Finally, the low pass filter removes the high frequency components and linearly outputs the Hall voltage.

### 3.1. Switched Hall Plate

The conventional two-phase spinning current circuit only consists of four N-channel Metal-Oxide-Semiconductor Field-Effect Transistor (MOSFET) NMOS and four P-channel MOSFET switches. Due to unbalanced equivalent Wheatstone bridge resistances of Hall plate and nonidealities of MOSFET switches, the output common mode voltage of the spinning current circuit is deviated from the center voltage of 1/2V_DD_, which seriously affects the reliable operation of the following-up amplifier. Therefore, the quiescent output voltage stability in 1/2V_DD_ is critical for improving the performance of the following signal conditioner.

**Figure 5:**
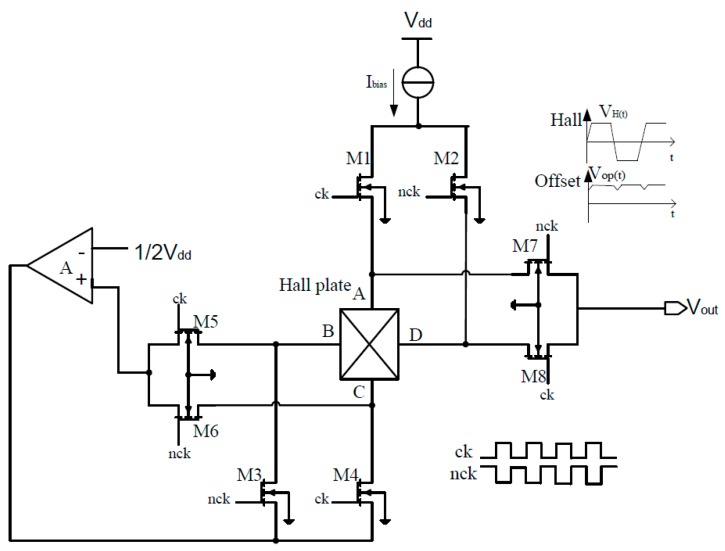
Circuit diagram of spinning current modulator of Hall sensor.

To solve this problem, a novel two-phase spinning current modulator is proposed, as shown in Figure 5. In addition to eight N-channel MOSFET switches M1~M8 controlled by two-phase complementary clocks ck and nck, it necessarily contains an operational amplifier. According to the concept of “virtual short” of the operational amplifier, the electric potential of the noninverting and inverting input terminals are equal. Thus, the common mode voltage of the operational amplifier is also equal to 1/2V_DD_. The NMOS switches M5~M8 are completely symmetrical, so the common mode quiescent output voltage of the spinning current modulation circuit clamps in 1/2V_DD_.

When ck level is high and nck level is low, the switches M1 and M4 turn on, and then the device bias current flows from the contact A to contact C. Consequently, the Hall voltage difference generates between the contact B and the contact D. At that moment, the transistors M5 and M8 turn on, and the output voltage is expressed by:

(3) $$V_{out}=\frac{1}{2}V_{H}+V_{OP}$$

When nck level is high and ck level is low, the switches M2 and M3 turn on, and then the bias current flows from the contact B to contact D, so the Hall voltage appears between the contact A and the contact C. At this time, the transistors M6 and M7 turn on, and the output voltage is given by:

(4) $$V_{out}=-\frac{1}{2}V_{H}+V_{OP}$$

It can be seen that the clocks ck and nck become high level in sequence and the current flow in the device changes from 0° to 90° state. The polarity of output Hall voltage *V_H_* changes, so Hall signal is modulated from the low frequency to high frequency. It is very interesting to note that the polarity of the offset voltage *V_OP_* remains quasi-constant during the spinning current course. As a result, the offsets coming from Hall device and instrumentation amplifier can be removed simultaneously.

### 3.2. Signal Conditioner

**Figure 6:**
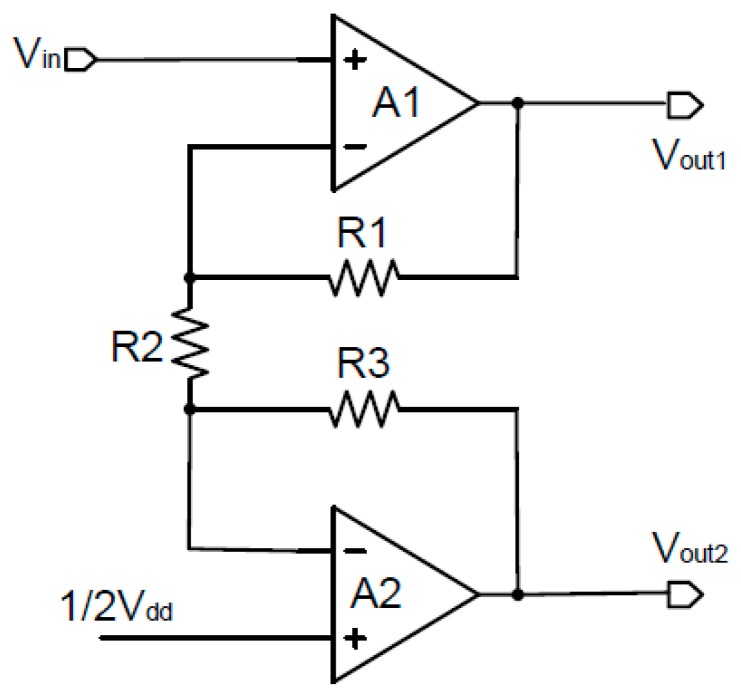
Instrumentation amplifier.

The signal conditioner includes two key components of the instrumentation amplifier and the demodulator. The instrumentation amplifier is a negative feedback loop amplifier that is composed of two basic operational amplifiers, as shown in Figure 6. It is dedicated to amplify Hall voltage and transports single-ended input signal into double-ended output signals. The closed loop gain of instrumentation amplifier is given by:

(5) $$A_{u1}=\frac{R_{1}+R_{3}}{R_{2}}+1$$

**Figure 7:**
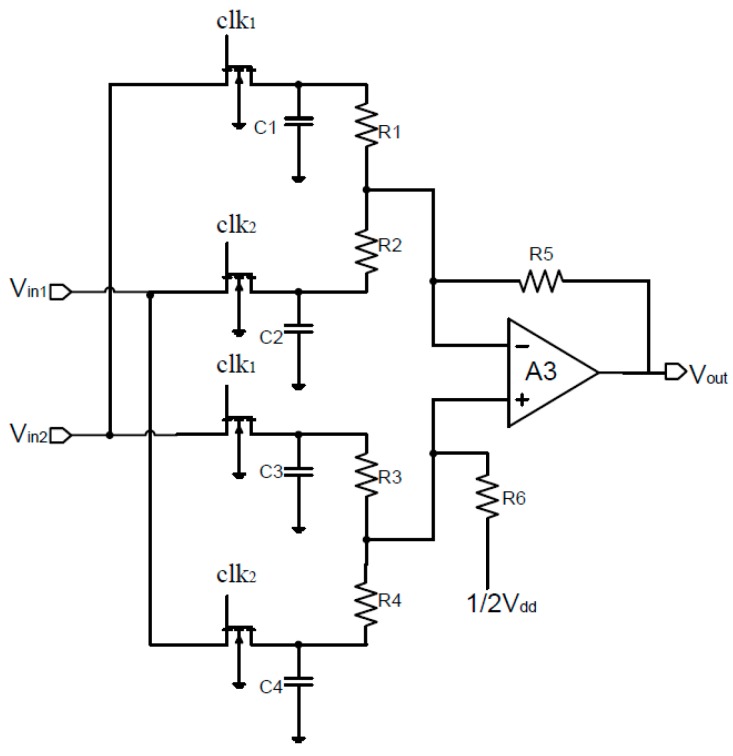
Demodulation circuit with sample/hold and adding functions.

Figure 7 shows the demodulator with S/H and adding functions [10]. When the common mode voltages of the S/H circuits are neglected, the output signals *V_A_* and *V_B_* of S/H circuits are expressed by [8,10]:

(6) $$V_{A}=\frac{1}{2}A_{u1}\left[V_{H}+V_{OP}+V_{OA}\right]$$

(7) $$V_{B}=\frac{1}{2}A_{u1}\left[V_{H}-V_{OP}-V_{OA}\right]$$

where, *A_u1_* and *V_OA_* are the gain and offset of the instrumentation amplifier.

Considering the quiescent output voltage *V_Q_* and ignoring the offset of the operational amplifier A_3_, we can obtain output voltage of adder circuit [8,10]:

(8) $$\begin{array}{l} V_{out}=V_{Q}+A_{u2}\cdot \left(V_{A}+V_{B}\right)\\ =V_{Q}+\frac{1}{2}A_{u1}\cdot A_{u2}\cdot V_{OP}(r)+A_{u1}\cdot A_{u2}\cdot V_{H} \end{array}$$

with *A_u__2_* the gain of adder and *V_OP_(r)* the residual offset voltage. If the *V_OP_(r)* is ignored, the Equation (8) is simplified as:

(9) $$V_{out}=V_{Q}+A_{u1}\cdot A_{u2}\cdot V_{H}=V_{Q}+A_{u(total)}\cdot V_{H}$$

Here, the total gain *A_u(total)_* of the signal conditioner is designed to about 46 dB. The gains of the instrumentation amplifier and the adder are 26 dB and 20 dB, respectively. Since the instrumentation amplifier also amplifies offset voltage, the gain of the instrumentation amplification cannot be too large.

It has been shown that the simple demodulator with S/H and adding function can eliminate the offset of the Hall device and amplifier, and modulate the Hall signal into low frequency.

### 3.3. Circuit Simulation

**Figure 8:**
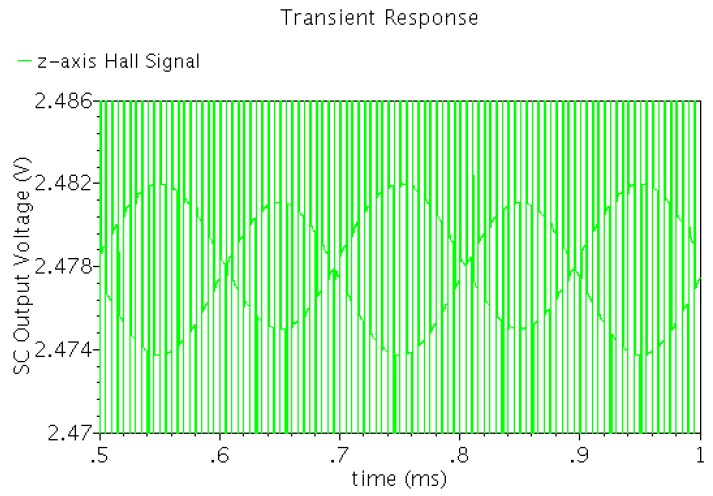
Transient output waveform of spinning current modulator of horizontal Hall sensor.

Based on the 0.8 μm HV CMOS technology, the circuit simulations of the integrated Hall sensor microsystem were performed. In order to obtain better temperature independence of the magnetic sensitivity, the Hall plate is biased in the constant current mode. A behavioral modeling of cross-like Hall plate is used for circuit simulation [19]. Here, the current related sensitivity of the Hall plate is 270 V/AT at 250 μA biasing current. Under the 5 V supply voltage, when the input clock frequency is 100 kHz and the magnetic field frequency is 5 kHz, the simulated transient output waveform of the spinning current modulator is shown in Figure 8. It is clearly observed that there is a 100 kHz modulated Hall signal appearing on a 2.477 V quiescent output voltage. In order to verify the ability of offset elimination of the Hall sensor microsystem, a 23 mV offset from Hall plate is superimposed on the 2.5 V quiescent output voltage. Actually, the spinning current modulator achieves a 2.5 V stable quiescent output voltage_._ Additionally, we observed that in the switch on/off transient, there generates transient spikes, which is due to the non-ideality of MOSFET switches.

**Figure 9:**
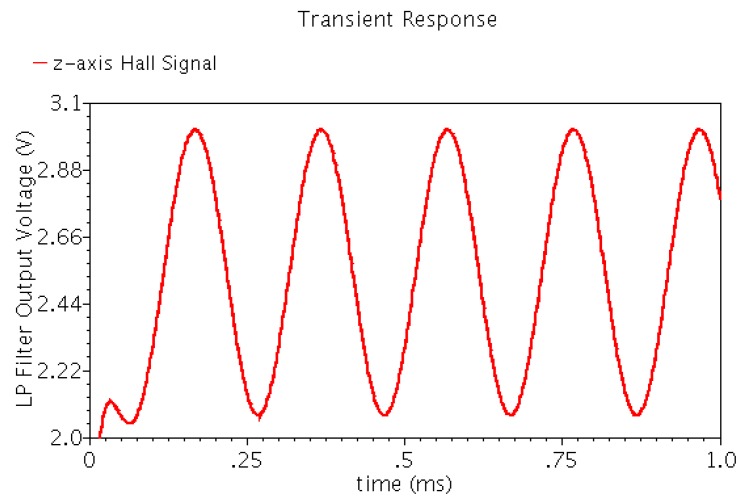
Transient output waveform of low-pass filter of the linear Hall sensor microsystem.

Figure 9 shows the final Hall output waveform after the low pass filtering. We found that the output waveform is smooth, and the harmonic component is very small. The output Hall voltage is linearly proportional to the magnetic flux density, which can accurately reflect the changes of the magnetic field.

## 4. Monolithic Hall Sensor Implementation

**Figure 10:**
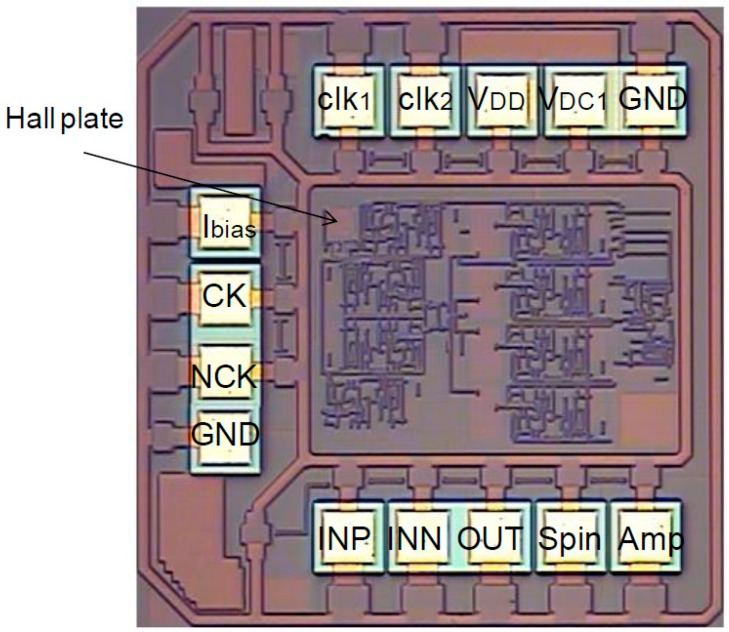
Micrograph of CMOS monolithic integrated Hall magnetic sensor.

A linear Hall sensor using dynamic offset cancellation technique was implemented in a monolithic chip by means of 0.8 μm HV CMOS technology. The Hall sensor chip, measuring 1 mm × 1 mm, is illustrated in Figure 10. The magnetic field used for the chip performance testing is produced by a ferromagnetic coil. When the coil is applied to the excitation current, a nearly uniform magnetic field is generated at the opening of the magnet. When we adjusted the excitation current of the ferromagnetic coil, the applied magnetic field is ranged from 0 to 200 mT. The Hall sensor is placed in this magnetic field for the Hall characteristic measurement.

**Figure 11:**
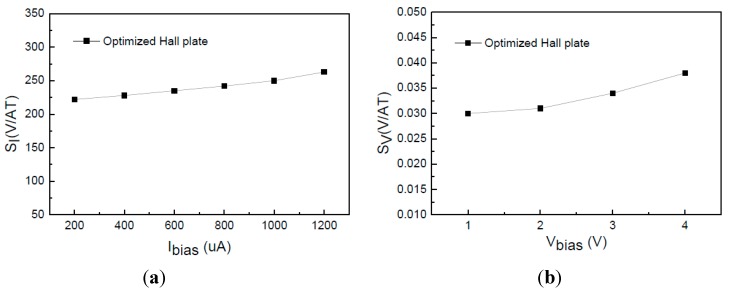
Tested magnetic sensitivity of cross-like Hall plate *versus* bias voltage or current. (**a**) *S_I_*; (**b**) *S_V_*.

**Figure 12:**
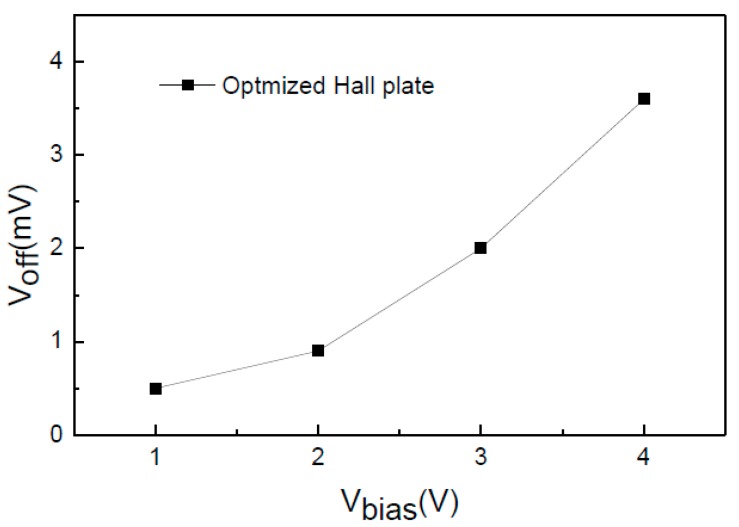
Tested offset voltage of cross-like Hall plate *versus* bias voltage.

First of all, we tested the magnetic sensitivity and offset voltage of the integrated cross-like Hall plate. Figure 11a,b show the *S_I_* and *S_V_* of the optimized Hall plates (*W* = *L* = 20 μm) *versus* bias current and voltage, respectively. It is seen that the *S_I_* and *S_V_* are increased with the biasing increasing, which is due to the junction field effect. When operating at the 1 mA biasing current, the measured *S_I_* is up to 250 V/AT. At 3 V biasing voltage, the *S_V_* reaches 0.034 V/VT. The optimized Hall plate obtains the high *S_I_* and *S_V_* simultaneously. Figure 12 shows the offset voltage of the optimized Hall plate. We can observe that the offset is apparently increased with the bias voltage increasing. At the 3 V bias voltage, the offset is as low as 2 mV.

**Figure 13:**
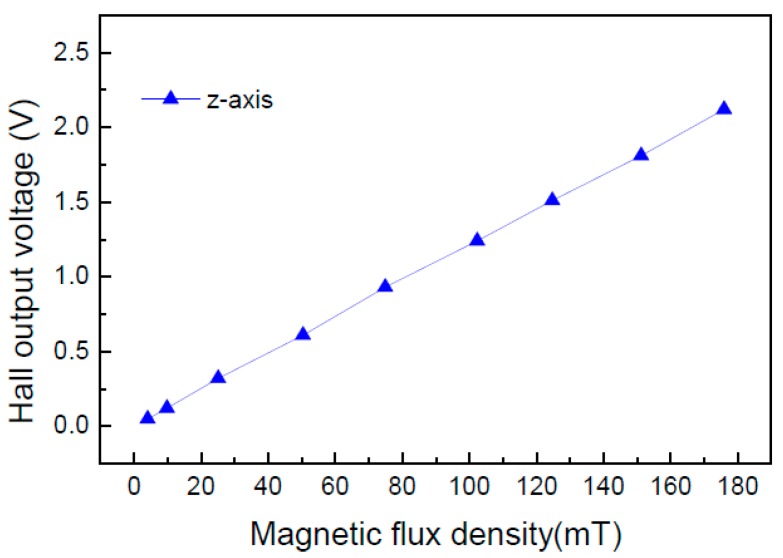
Hall output voltage of the integrated Hall sensor *versus* magnetic flux density.

Next, we tested the performances of the monolithic Hall sensor microsystem at the 5 V supply voltage. The input clock frequency is 100 kHz and the Hall plate is biased at 250 μA. Figure 13 shows the relationship of Hall output voltage *versus* magnetic flux density. When the magnetic flux density exceeds ±5 mT, the output Hall voltage linearly increases with the magnetic flux density. When the magnetic field is larger than ±175 mT, the output Hall voltage is saturated. This is because the output voltage is close to the supply voltage. In the magnetic flux density range from ±5 mT to ±175 mT, the linearity of the output Hall voltage is higher than 99%, which shows that the integrated Hall sensor has strong ability to linearly amplify a weak Hall signal and eliminate large offset.

**Figure 14:**
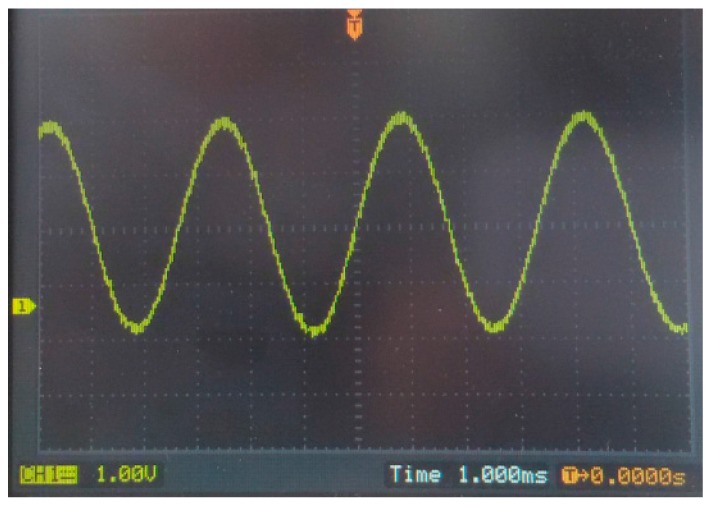
Transient output waveform of the integrated Hall sensor for a 1 kHz 170 mT sinusoidal magnetic flux density.

Figure 14 shows the Hall sensor transient output voltage for a 1 kHz 170 mT sinusoidal magnetic flux density. The output waveform is proportional to the change of magnetic field, and the nonlinear distortion is very small.

Table 1 summarizes the tested performances of the monolithic integrated Hall sensor microsystem and makes comparisons with other two reported Hall sensor chips. At the 5 V supply, the static power consumption is 20 mW, the maximum Hall output voltage reaches ±2.1 V, and the output equivalent residual offset is 0.48 mT. Reference [10] presented a 5-V monolithic linear Hall sensor using a 2 μm conventional Bipolar CMOS (BiCMOS) technology. It can be seen that our work achieves almost the same performances with the work reported in the literature [10] using the low cost CMOS process. Although the linearity of Hall output voltage in our work is slightly less than that in the literature [10], our work achieves the larger magnetic field measurement range. Moreover, our work obtains smaller static power consumption and residual offset voltage. Reference [2] also presented a linear Hall sensor for current measurement fabricated in 0.8 μm CMOS process. It is noticed that the magnetic field measurement range is only ±50 mT in the literature [2], which is obviously smaller than our results. In our work, the linearity is still larger than 99% across the maximum ±175 mT magnetic field measurement range.

**Table 1:** Performance summary and comparison of CMOS monolithic integrated Hall sensors.

Parameters	Reference[10]	Reference[2]	This Work
Technology	2 μm BiCMOS	0.8 μm CMOS	0.8 μm HV CMOS
Supply voltage	5 V	5 V	5 V
Static power consumption	35 mW	N/A	20 mW
Quiescent working point	2.5 V	N/A	2.5 V
Measurement range	±100 mT	±50 mT	±175 mT
Equivalent residual offset	0.5 mT	N/A	0.48 mT
linearity	99.9%	>99%	>99%

## 5. Conclusions

A monolithic integrated Hall sensor is implemented using a 0.8 μm high voltage CMOS process. The chip integrated with a highly sensitive cross-like Hall plate realizes the effective elimination of offset voltage and linear amplification of a weak Hall signal by means of an improved switched Hall plate and a simple signal conditioner. The device measuring results show that the offset voltage of the optimized Hall plate is 2 mV at the 3 V biasing voltage. The *S_I_* and *S_V_* achieve 250 V/AT at 1 mA biasing current and 0.034 V/VT at the 3 V biasing voltage, respectively. At the 5 V supply, the Hall sensor microsystem testing results show that the linearity of Hall output voltage is larger than 99% in the magnetic flux density range from ±5 mT to ±175 mT. The total static power consumption is 20 mW, and the output equivalent residual offset is 0.48 mT.
